# Initial Weight Loss, Anthropometric Parameters, and Proinflammatory Transcript Levels in Patients with Class I Obesity

**DOI:** 10.3390/biomedicines11082304

**Published:** 2023-08-18

**Authors:** Beata Jabłonowska-Lietz, Grażyna Nowicka, Marta Włodarczyk, Sławomir Rejowski, Maria Stasiowska, Małgorzata Wrzosek

**Affiliations:** 1 Medical Center, National Institute of Public Health NIH—National Research Institute, 24 Chocimska St., 00-791 Warsaw, Poland; 2 Department of Biochemistry and Pharmacogenomics, Center for Preclinical Research, Medical University of Warsaw, 1 Banacha St., 02-097 Warsaw, Poland; 3 Liver and Internal Medicine Unit, Department of General, Transplant and Liver Surgery, Medical University of Warsaw, 1A Banacha St., 02-097 Warsaw, Poland; 4 Department of Anaesthesia and Intensive Care, University College London Hospital, National Hospital for Neurology and Neurosurgery, Queen Square, London WC1N 3BG, UK

**Keywords:** weight loss predictors, subcutaneous adipose tissue, *FTO* rs9930506 polymorphism, proinflammatory gene expression, BIA, HOMA-IR

## Abstract

Research into early predictors of effective weight loss could help determine more effective therapeutic interventions. In this study, 106 subjects with class I obesity, genotyped with the fat mass and obesity-associated (*FTO*) rs9930506 gene variant, were enrolled into a 12-week weight loss program (WLP). Anthropometric and body composition measurements were controlled with bioelectrical impedance analysis (BIA) at baseline and after 4 and 12 weeks. Biopsies of abdominal subcutaneous adipose tissue (AT) and venous blood samples were collected to monitor changes in interleukin 6 (*IL-6*), tumor necrosis factor-alpha (*TNF-α*), and nuclear factor kappa B (*NF-κB*) mRNA levels in white blood cells (WBCs) and to assess if changes in WBC gene expression reflected changes in adipose tissue. The *FTO* rs9930506 variant had no effect on weight loss and no reduction in proinflammatory transcripts in WBCs or AT. Changes in anthropometric parameters were associated with changes in carbohydrate metabolism. A linear regression model showed that initial weight loss (after 4 weeks of the WLP) was the most predictive factor of weight loss success after 12 weeks of the WLP. Changes in plasma lipids or proinflammatory transcript levels in WBCs or AT were not associated with weight loss effectiveness. However, the gene expression in WBCs did reflect changes occurring in subcutaneous AT.

## 1. Introduction

Excess adiposity is the consequence of a positive energy balance when energy intake exceeds energy expenditure. This results predominantly from unhealthy lifestyle factors which include a high caloric diet and/or low physical activity [1]. The effectiveness of most weight loss therapeutic interventions is relatively low, and most programs are unable to reverse the increasing prevalence of obesity. Research looking for early predictors of effective weight loss could help patients with the high-risk genotypes of the fat mass and obesity-associated (*FTO*) gene who have difficulty with effective weight loss achieve better treatment results. It is recognized that allelic variants of the *FTO* gene, i.e., rs9939506, affect fat deposition, fat mass percentage [2], waist circumference, hip circumference, and body mass index (BMI) [2,3,4]. Our study on Polish adults found a significant association between the *FTO* rs9930506 GG genotype and higher BMI [5]. A similar correlation was reported in Italian and Sardinian individuals [4,6], whilst a metanalysis by Doaei et al. [3] showed that the G allele of the *FTO* rs9930506 variant in the European population was associated with a higher obesity risk. The effect on anthropometric parameters is similar to that on other (biochemical) obesity-related parameters. This can be seen in individuals with the risk allele (G) of the *FTO* rs9930506 variant who have lower concentrations of the high density lipoprotein (HDL) cholesterol [2,4,7] and higher levels of systemic inflammatory markers such as C-reactive protein (CRP) [2]. Conversely, many coexisting factors, including biochemical, metabolic, and environmental indicators, can have an impact on achieving and maintaining effective weight loss [8,9,10]. For instance, individuals with larger reductions in fasting insulin and insulin resistance after a 6-week dietary intervention had lower weight regain after the 6-week stabilization period [11]. Likewise, greater changes in fasting insulin induced by an 8-week low-calorie diet (LCD) were associated with lower weight regain in the 6-month weight maintenance phase [10]. Furthermore, an increase in triglyceride concentrations at a 9-month follow-up after dietary intervention was linked to higher weight regain and fat expansion [12]. On the other hand, lower baseline leptin concentration [13], higher baseline adiponectin, and a larger reduction in leptin concentration after a 6-moth dietary intervention [14] have been associated with successful weight loss. It has been proposed that difficulties in losing weight result mainly from adipose tissue remodeling and are associated with a higher number of stromal vascular cells, increased cell stress, reduced lipolysis, and altered adipokine secretion [9,15]. Conversely, fat mass reduction is associated with the recruitment of new and small adipocytes and improves insulin sensitivity [10,16]. Thus, body weight reduction plays a key role in preventing type 2 diabetes [17,18] and many other obesity-associated diseases [19,20,21]. Excessive fat accumulation is associated with chronic systemic low-grade inflammation and inflammatory cell infiltration in adipose tissue (AT) [22]. The overexpression of proinflammatory genes in the adipose tissue plays a key role in the pathogenesis of obesity and the development of obesity-related metabolic disturbances [23,24]. The nuclear factor kappa B (*NF-κB*) pathway and the overexpression of tumor necrosis factor-alpha (*TNF-α*) and interleukin 6 (*IL-6*) genes affect adipocyte biology by promoting adipose tissue dysfunction and impairing insulin sensitivity [25]. A reduction in body fat because of weight loss interventions can be beneficial in reducing obesity-related inflammation. A decrease in inflammatory markers such as plasma concentrations of circulating IL-6, TNF-a, MCP1 (monocyte chemoattractant protein-1), and CRP was observed in previous clinical trials [11,26,27,28,29]. It may be associated with a change in the expression of appropriate genes in white blood cells (WBCs) that are part of the immune system. However, it is not known whether and to what extent changes in the expression of proinflammatory genes in adipose tissue are related to changes in the expression of these genes in WBCs. Little is known about the potential effect of proinflammatory gene expression in adipose tissue and white blood cells (WBCs) and its effect on weight reduction efficacy. There is also a lack of data regarding the association between the *FTO* rs9930506 polymorphism and changes in proinflammatory gene expression due to body weight loss. Therefore, in this study, changes in *NF-κB*, *IL-6*, and *TNF-α* mRNA levels were assessed at baseline and at 4 and 12 weeks after a weight loss program. Previous studies have looked at these parameters after 12 weeks [30,31] or even longer periods of observation [28,32,33]. This study looks at whether the initial period of weight loss (4 weeks) affects proinflammatory gene expression. The primary objective of this study was to determine the impact of the risk GG genotype of the *FTO* rs9930506 variant on baseline parameters and changes in anthropometric and metabolic parameters in response to a 12-week weight loss program (WLP). We also evaluated the effect of weight loss after 4 and 12 weeks of the WLP on anthropometric and metabolic parameters and changes in the *NF-κB*, *TNF-α*, and *IL-6* mRNA levels in white blood cells (WBCs) and subcutaneous adipose tissue (AT) of the study subjects. Additionally, we looked at whether gene expression in WBCs reflects changes occurring in the subcutaneous AT in response to a 12-week weight loss program and its effectiveness on body weight loss.

## 2. Materials and Methods

### 2.1. Study Population

A total of 130 individuals with obesity were recruited from patients who had been referred to the Outpatient Clinic at the National Food and Nutrition Institute in Warsaw for obesity treatment or routine general health screening. All recruited participants underwent a comprehensive medical evaluation including clinical history assessment, physical examination, fasting blood testing, anthropometric parameters, and blood pressure measurements. Inclusion criteria included age 30–50 years, male and female sex, BMI between 30.0–34.9 kg/m^2^ (which is classified as class I obesity as per the World Health Organization classification [34]), weight stable (body mass changes <±3 kg of current body weight) for at least 3 months, and non-smokers (for at least 5 years). Exclusion criteria included pregnancy or lactation, history of bariatric surgery, anorexia nervosa and bulimia, taking anti-inflammatory drugs, medications, or dietary supplements known to support obesity treatment or influence plasma lipid concentration, type 1 or 2 diabetes, history of hyperglycemia and hyperlipidemia treatment, endocrine disorders (e.g., thyroid and parathyroid disorders, Cushing’s syndrome, and polycystic ovary syndrome), renal and hepatic disorders, autoimmune diseases, cancer, and implanted pacemakers or other metal implants. From the initially selected subjects who agreed to participate in this study and provided informed consent, 24 individuals were excluded due to insufficient medical documentation or exclusion criteria. The final cohort of 106 subjects who were enrolled in a 12-week weight loss trial had their *FTO* gene rs9930506 polymorphism tested. A control group of 268 individuals with similar characteristics such as age and sex but normal BMI (18.5–24.9 kg/m^2^) were also recruited to compare the genotype and allele frequencies of the studied genetic variant. They were healthy individuals without chronic metabolic and endocrine diseases. Patients who missed any part of the follow-up regime were excluded from the final analyses. A total of 76 participants underwent a complete set of biochemical and genetic tests as well as anthropometric measurements to monitor changes resulting from the weight loss intervention applied. In addition, 20 participants (65% of whom were women) volunteered to undergo a subcutaneous abdominal adipose tissue biopsy at baseline and after 12 weeks of intervention.

### 2.2. Study Design

**Figure 1:**
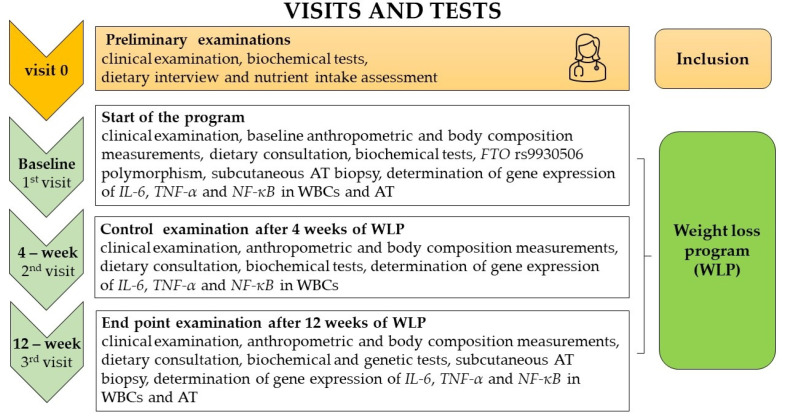
The timeline of the 12-week weight loss intervention.

The weight loss program (WLP) was conducted from 2012 to 2016 in 106 individuals. The timeline of the WLP is summarized in Figure 1. The following time points were proposed: baseline (1st visit), after 4 weeks (2nd visit), and after 12 weeks from baseline (3rd visit, the end point of the WLP intervention program). The weight loss, anthropometric, and body composition measurements were analyzed at each time point of the study (Figure 1). The frequency of *FTO* rs9930*5*06 genotypes in the study population and controls was assessed.

### 2.3. Weight Loss Intervention

The subjects participated in a 12-week weight loss program with a balanced low-calorie diet (LCD). According to the WHO and Polish recommendations, the diet took 50–55% of total energy intake (TEI) from carbohydrates (<10% of energy from sucrose), 25–30% of TEI from fat, and 15–20% of TEI from protein. To estimate the total energy expenditure (TEE), the basal metabolic rate (BMR), estimated by applying the Mifflin equation [35], was corrected by the physical activity level [36]. The calorie intake was reduced by 25–30% of that required to maintain a stable weight. Dietary intake assessment was monitored with a 3-day food record (which consisted of 2 non-sequential weekdays and 1 weekend day) and conducted by qualified dietitians. The recorded data were analyzed using the Diet 5 software (version 5.0, National Food and Nutrition Institute, Warsaw, Poland) [37] based on Polish Food Composition Tables [38]. Data on eating habits were collected and consulted based on a proprietary nutritional questionnaire. “Face-to-face” standardized dietitian counselling and education was conducted monthly in an outpatient clinic. Additionally, telephone- and e-mail-based guidance was conducted. Participants were advised to follow an individual well-balanced diet based on the patient’s food preferences, e.g., recommended meal pattern consisted of 3 meals and 2 snacks daily (including hunger and satiety signals) with emphasis on fruit and vegetables, whole-grain products, and sources of n-3 fatty acids and legumes to reduce animal protein. Ad libitum water intake was recommended. In addition to dietary guidelines, patients were also advised to raise physical activity to a minimum of 150 min/week [39]. Moreover, patients were informed about the role of other factors related to a healthy lifestyle (including proper eating habits or adequate sleep) in reducing and maintaining a stable body weight. Patients were also advised that weight loss could significantly reduce the risk of obesity-related complications.

### 2.4. Anthropometric Measurements

Anthropometry, including body weight, waist circumference (WC), and hip circumference (HC), was assessed using standardized techniques and equipment. Body weight (at each visit) and body height (at baseline) were measured in underwear with accuracies of 0.1 kg and 0.5 cm, respectively. The waist circumference measurement was taken at the midpoint between the lower margin of the rib cage and the top of the iliac crest, while the hip circumference was measured around the widest portion of the buttocks (with the flexible tape parallel to the floor). The following parameters of body composition were obtained: body fat mass (FM, kg), fat mass percentage (FM, %), and visceral fat level (VFAT, 1–18 scores) assessed by the bioelectrical impedance analysis (BIA) method using the 8-electrode TANITA MC 180 MA camera (TANITA Corporation, Tokyo, Japan) according to built-in algorithms. The fat-free mass (FFM) refers to all the body components (including total body water, muscle mass, and bone mass) except fat mass.

Measurements were taken in the morning, after overnight fasting, on the same day, or the day before biopsy and blood sampling. GMON Tanita professional software (version 3, TANITA Corporation, Tokyo, Japan) was used for the analysis. Based on anthropometric measurements, the BMI and waist–hip-ratio (WHR) indexes were calculated. Alternative parameters of body fat distribution—BAI (body adiposity index) and WHtR (waist-to-height ratio)—were calculated and are described in more detail in our previous paper [40]. Obesity was classified according to the World Health Organization criteria [41].

### 2.5. Measurements of Biochemical Parameters

Venous blood samples (10 mL) were collected after overnight fasting from all patients before, at the 2nd, and at the 3rd visit to monitor biochemical changes that occur during weight reduction. The serum concentration of biochemical parameters, including total cholesterol (TC), high-density lipoprotein (HDL) cholesterol, triglycerides (TG), glucose, and insulin, was determined using automatic analyzers in a certified laboratory for clinical biochemistry. The low-density lipoprotein (LDL) cholesterol level was calculated using the Friedewald formula [42]. The adiponectin, leptin, and high-sensitivity C-reactive protein (CRP-hs) concentrations were evaluated using a commercially available enzyme-linked immunosorbent assay (ELISA) (Immundiagnostik AG, Bensheim, Germany). Insulin resistance was assessed by the Homeostasis Model Assessment for Insulin Resistance (HOMA-IR) index using the following formula: HOMA-IR = [fasting plasma glucose (nmol/L) × fasting plasma insulin (µU/mL)/22.5] [43].

### 2.6. Genotyping of the FTO rs9930506 Polymorphism

Genomic DNA was extracted from peripheral whole blood (1 mL) using the Blood Mini genomic DNA purification kit from A&A Biotechnology according to the manufacturer’s instructions. The DNA concentration and purity were determined with UV spectrophotometry, measuring absorbance ratios of 260 nm/280 nm. The genotyping of the *FTO* rs9930506 polymorphism was performed by TaqMan allelic discrimination real-time PCR. A validated TaqMan SNP genotyping assay was obtained from Life Technologies. The initial step of the allelic discrimination genotyping assay protocol consists of 95 °C for 10 min, followed by 92 °C for 15 s and 60 °C for 1 min, with steps 2–3 repeated for 40 cycles. More than 50 percent of the studied genotypes were determined twice, and genotyping was 100% concordant.

### 2.7. Adipose Tissue Biopsy

Abdominal subcutaneous AT needle biopsies (approximately 1 g) were collected 6 to 8 cm laterally from the umbilicus under local anesthesia (2% lidocaine) after overnight fasting at baseline and after 12 weeks of the WPL. The AT samples were immediately rinsed with sterile saline [44], snap-frozen in liquid nitrogen, and stored at −80 °C until analysis. Subcutaneous adipose tissue biopsies were performed in 20 paired patients at baseline and after 12 weeks of the WLP. An abdominal adipose tissue biopsy was performed in the Clinic for Surgery and Organ Transplantation at the Medical University of Warsaw by a medical doctor with prior experience of conducting minor surgery. The study protocol was conducted in accordance with Declaration of Helsinki guidelines and approved by the Institutional Bioethics Committees at the National Food and Nutrition Institute (no. NZ7/04559/10/2010 and NZ7/04559/10/2012) and at the Medical University of Warsaw (no. KB/127/2012 and KB/67/2017). All registered participants signed an informed consent form after receiving verbal and written information about the study’s objectives and methodology.

### 2.8. Determination of the Gene Expression of IL-6, TNF-α, and NF-κB in the Patients’ Blood and Subcutaneous Adipose Tissue

Total RNA was extracted from blood using a Total RNA Mini kit (A&A Biotechnology, Gdynia, Poland) and from subcutaneous AT using an RNeasy Lipid Tissue Mini Kit (Qiagen, Hilden, Germany). The concentration and purity of RNA were evaluated with a micro-volume UV-Vis spectrophotometer (Quawell Q3000, Quawell Technology Inc., San Jose, CA, USA). RNA was reverse transcribed to cDNA with a High-Capacity RNA-to-cDNA Kit (Applied Biosystems, Foster City, CA, USA). The expressions of *IL-6*, *TNF-α* and NF-κB genes were determined with quantitative real-time PCR (qRT-PCR) using TaqMan Gene Expression Assays (Table 1, Thermo Fisher Scientific Inc., Waltham, MA, USA) and TaqMan™ Gene Expression Master Mix (Thermo Fisher Scientific Inc., Waltham, MA, USA). Analyses were performed using a ViiA™7 Real-Time PCR system (Applied Biosystems; Thermo Fisher Scientific, Inc., Waltham, MA, USA). All samples were run in triplicate and average values were calculated. The data were normalized to the reference gene (*18S*, Table 1), as its expression was the most stable compared with the few possible house-keeping genes tested, and the fold change between the mRNA levels of each target gene compared with the control group was expressed as 2^−ΔΔCt^, where ΔΔCt = (Ct target − Ct *18S* rRNA) studied group − (Ct target − Ct *18S* rRNA) control group (Ct, cycle threshold) [45].

### 2.9. Statistical Analyses

Statistica 13.0 (StatSoft, Tulsa, OK, USA) was used to conduct the statistical analysis of the results. A *p* value < 0.05 was considered statistically significant. The quantitative variables were reported as mean ± standard deviation (SD). The normality of distribution was verified with the Shapiro–Wilk test. The analysis of variance (ANOVA) and/or Kruskal–Wallis tests were used to assess the differences for studied parameters. The differences between the two groups were assessed using the Mann–Whitney U nonparametric tests or Student’s *t*-test. Univariate comparisons were made between the groups using the χ^2^ test for categorical variables. Spearman’s test was used to examine associations between anthropometric and biochemical parameters at baseline, after 4 weeks, and after 12 weeks of the WLP. Finally, a linear regression analysis was performed to determine the influence of covariates that may independently affect weight loss in response to the 12-week WLP. To further describe these associations, a multiple linear regression model was fitted to test whether the variables studied were able to predict the final change in body weight (ΔBW) after an adjustment for each parameter depending on the analysis. The regression variables included sex and age as fixed effects. A stepwise variable selection based on AIC (the Akaike information criterion) was used to choose a model. The regression model results are presented as regression coefficients (β) and 95% confidence intervals (CI) for regression coefficients.

## 3. Results

### 3.1. The Effects of the FTO rs9930506 Polymorphism on Body Weight and Composition and Biochemical and Proinflammatory Parameters (at Baseline and throughout the Intervention)

The study group consisted of 106 individuals (72 women and 34 men) with a mean age of 38.7 ± 5.5 years and an average BMI of 32.6 ± 2.3 kg/m^2^. The patients reported consuming 2125.6 ± 771.0 (kcal) per day and their duration of obesity (estimated by subtracting the current age from the age of obesity onset) was 15.3 ± 8.1 years. The baseline characteristics of the whole study group are presented in Table 2. There was no significant difference in BMI between male and female participants. However, statistically significant higher values of waist circumference (WC), visceral fat level (VFAT), and fat-free mass (FFM) were observed in male versus female patients. The latter have higher fat mass percentage (FM%), hip circumference (HC), and BAI in comparison with men. All participants had their *FTO* rs9930506 polymorphism and baseline characteristics analyzed, the results of which are presented Table 2. Genotype distribution was similar in both women and men: 29% vs. 32% for the GG genotype, 49% vs. 50% for the AG genotype, and 22% vs. 18% for the AA genotype, respectively (χ^2^ = 0.33; *p* = 0.85). We did not find significant differences in body weight, BMI, fat mass percentage, VFAT, fasting glucose, insulin, HOMA-IR, total cholesterol, triglycerides, hs-CRP, leptin, and adiponectin between the three genotypes of the *FTO* rs9930506 polymorphism (Table 2). Such interactions were not found on subsequent visits or according to sex.

The prevalence of genotypes and alleles within the investigated *FTO* gene polymorphism in the study population (class I obesity, BMI between 30–35) undergoing a dietary intervention is shown in Table 3. To estimate the risk genotype frequency a comparison was made between the genotype and allele prevalence observed in the study group and in the age- and sex-matched control group with a normal bodyweight (BMI between 18.5–24.9 kg/m^2^) consisting of 268 individuals. The distribution of the genotypes of *FTO* rs9930506 presented in Table 3 did not deviate from Hardy–Weinberg equilibrium in either the study group (*p* = 0.92; χ^2^ = 0.01; df = 1) or the control group (*p* = 0.06; χ^2^ = 3.65; df = 1). The prevalence of the rs9930506 G allele among the study subjects with obesity was higher than among the controls (55% vs. 44%, OR = 1.49, *p* = 0.014). The statistical analysis (Table 3) revealed a significant association between the polymorphism rs9930506 and obesity in the recessive (OR = 2.09; *p* = 0.006) and co-dominant models of inheritance (OR = 1.54; *p* = 0.011).

### 3.2. Relation between the Effectiveness of Weight Loss Program and Changes in Clinical Parameters

All biochemical and anthropometric measurements were successfully performed in 76 participants to monitor changes resulting from the WLP. At the end of the intervention, 55% of participants achieved moderate weight loss, i.e., a ≥5% reduction in initial body weight, and these subjects were classified as successful weight losers (group A). The remaining study subjects with a weight reduction < 5% were included in group B. In group A, the body weight reduction (ΔBW) of −8.3 ± 3.0 kg was significantly higher than in group B (−2.1 ± 1.2 kg; *p* < 0.001) at the end point of the WLP.

The differences between each visit (weeks 4 and 12) and baseline (visit 1) among individuals who lost at least 5% of their initial body weight (group A) and those who lost less than 5% of their initial body weight (group B) are summarized in Table 4. At baseline, the mean BMI in group A was 32.2 ± 2.3. There was no statistically significant difference in BMIs between group A and B (32.7 ± 2.3, *p* = 0.435) or any other anthropometric and biochemical parameters. Initial weight loss (∆_2-1_ BW after 4 weeks) did not cause significant differences between the groups for the ∆_2-1_ BAI and ∆_2-1_ HOMA-IR. At the end point of the dietary intervention, body weight and BMI decreased in both groups (A and B). This reduction was three times higher in group A versus B (Table 4). Similarly, the reductions in FM % (*p* < 0.001), FM kg (*p* < 0.0001), WC (*p* < 0.0001), and VFAT, as estimated by BIA (*p* < 0.001, were three times higher in group A versus group B (Table 4).

Table 4 highlights the difference in changes in clinical measures between men and women. In the studied group, men had higher body weight reduction after 12 weeks (∆_3-1_ BW; −7.08 ± 4.1 vs. −4.8 ± 3.6 kg, *p* = 0.031) and higher visceral fat level reduction after 4 and 12 weeks compared with women (∆_2-1_ VFAT: −1.0 ± 0.9 vs. −0.6 ± 0.6, *p* < 0.001 and ∆_3-1_ VFAT: −1.8 ± 1.1 vs. −0.8 ± 0.9, *p* < 0.001, respectively). In addition, in group A with weight loss ≥ 5%, men had higher visceral fat level reduction after 4 and 12 weeks than women (∆_2-1_ VFAT: −1.4 ± 0.8 vs. −0.6 ± 0.6, *p* = 0.001 and ∆_3-1_ VFAT: −2.3 ± 1.1 vs. −1.3 ± 0.8, *p* = 0.004, respectively, Table 4). Further analysis revealed greater initial weight loss (∆_2-1_ BW after 4 weeks) in men (∆_2-1_ BW = −6.1 ± 2.4 kg) than in women (∆_2-1_ BW = −4.3 ± 1.9 kg; *p* = 0.014). We also found greater initial fat-free mass loss (∆_2-1_ FFM) in men versus women (∆_2-1_ FFM = −2.8 ± 1.2 kg vs. −1.6 ± 1.8 kg, respectively; *p* = 0.040).

There were no differences in parameter changes between men and women in the group with lower body weight loss (group B, weight loss < 5%).

### 3.3. The Effects of a Dietary Intervention on White Blood Cells (WBCs) and Subcutaneous Adipose Tissue (AT) Proinflammatory Gene Expression

**Figure 2:**
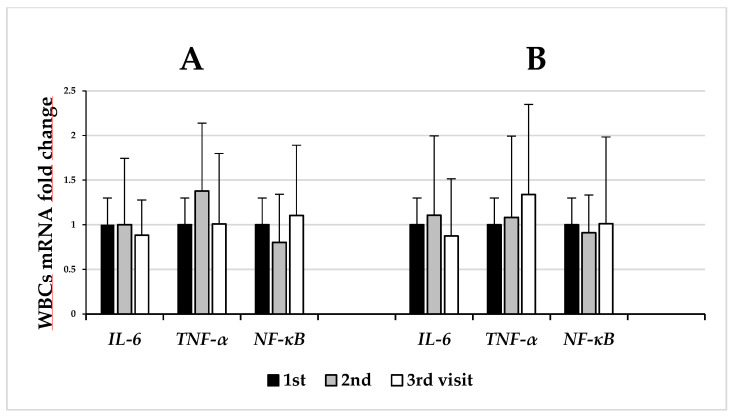
The effects of weight loss on the *IL-6*, *TNF-α*, and *NF-κB* mRNA levels in white blood cells (WBCs) among individuals with a higher (≥5%) weight loss ((**A**), *n* = 40) and with a lower (<5%) weight loss ((**B**), *n* = 36). Time points: baseline, after 4 weeks (2nd visit), and after 12 weeks (3rd visit) of the weight loss program. Fold changes were evaluated using the 2^−ΔΔCt^ method, showing the comparison between each time point and the baseline. The data represent mean and SD.

Changes in the *IL-6*, *TNF-α*, and *NF-κB* mRNA levels in the patients’ white blood cells were assessed at baseline and after 4 and 12 weeks of the weight loss program (Figure 2).

The results were shown among individuals with a higher (≥5%) weight loss (group A, *n* = 40) and with a lower (<5%) weight loss (group B, *n* = 36).

The higher reduction in body weight in group A did not result in a significant decrease in the *IL-6*, *TNF-α*, and *NF-κB* gene expression during and after the 12-week WLP (second and third visits) (Figure 2A). In group B, among participants with a lower weight loss (<5%), no statistically significant differences were observed in the levels of *NF-κB*, *IL-6*, and *TNF-α* gene expression after 4 weeks (second visit) or 12 weeks (third visit) of the weight loss program (Figure 2B).

**Figure 3:**
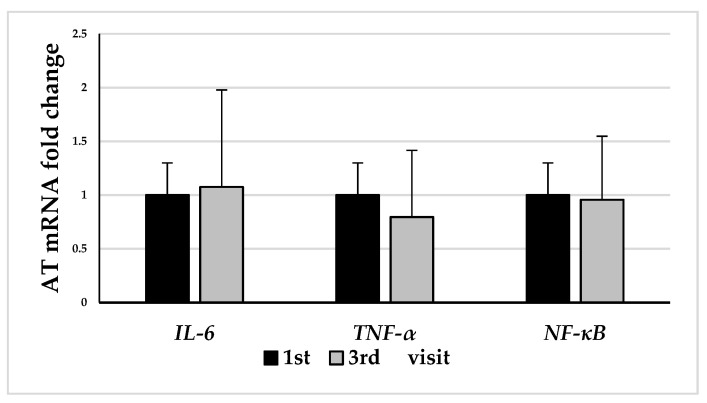
The effects of weight loss on the *IL-6*, *TNF-α*, and *NF-κB* mRNA levels in subcutaneous adipose tissue (AT) (*n* = 20). Fold changes were evaluated using the 2^−ΔΔCt^ method, showing the comparison between the end point of the 12-week weight loss program and the baseline. The data represent mean and SD.

Among 20 patients who consented to biopsy of adipose tissue, 65% of whom were women, a subcutaneous abdominal adipose tissue biopsy was performed at baseline and after 12 weeks of the weight loss program. The reduction in body weight (∆_3-1_ BW) by −9.0 ± 4.7% was accompanied by a decrease in fat mass percentage (∆_3-1_ FM %) of 5.5 ± 3.6% in response to the 12-week weight loss program. Changes in *IL-6*, *TNF-α*, and *NF-κB* mRNA levels in patients’ subcutaneous adipose tissue induced by the 12-week weight loss program are shown in Figure 3.

We did not find a statistically significant reduction in *IL-6*, *TNF-α*, and *NF-κB* gene expression in adipose tissue at the end of the weight loss program compared with baseline levels (Figure 3). Subsequently, it was assessed whether gene expression in the study subjects’ WBCs reflected changes occurring in their subcutaneous AT. Table 5 shows the correlation between changes in proinflammatory gene expression in patients’ white blood cells and subcutaneous adipose tissue in response to the 12-week weight loss program. The gene expression changes (∆_3-1_) were calculated by subtracting the final levels from the baseline value. It was found that the ∆_3-1_ AT *NF-κB* was strongly correlated with the ∆_3-1_ AT *IL-6* (r = 0.84, *p* < 0.001) and the ∆_3-1_ AT *TNF-α* (r = 0.85, *p* < 0.05), as well as with the ∆_3-1_ WBC *NF-κB* (r = 0.75, *p* < 0.05). On the other hand, the ∆_3-1_ WBC *NF-κB* was most strongly correlated with the ∆_3-1_ AT *IL-6* (r = 0.89, *p* < 0.001). There were no statistically significant correlations between the gene expression changes in WBCs.

### 3.4. Predictors of Successful Weight Loss and Improvement in Metabolic Measurements

A correlation analysis was used to identify variables strongly associated with changes in anthropometric parameters after 12 weeks of the weight loss program (Table 6). Changes in biochemical and anthropometric variables after weight loss were calculated by subtracting the final levels from the corresponding ones at baseline.

Body weight changes (represented by ∆_3-1_ BW and ∆_3-1_ BMI) positively correlated with anthropometric measures (∆_3-1_ WC, ∆_3-1_ HC, ∆_3-1_ WHR, ∆_3-1_ BAI, and ∆_3-1_ WHtR) and with the following biochemical parameters: ∆_3-1_ glucose, ∆_3-1_ insulin, and ∆_3-1_ HOMA-IR. There was no significant correlation between body weight changes and the following parameters: ∆_3-1_ TG, ∆_3-1_ total cholesterol, ∆_3-1_ HDL cholesterol, ∆_3-1_ LDL cholesterol, ∆_3-1_ CRP, ∆_3-1_ adiponectin, and ∆_3-1_ leptin (Table 6). As shown in Table 6, the ∆_3-1_ BW correlated the most with the ∆_3-1_ WC (r = 0.72, *p* < 0.001), ∆_3-1_ WHtR (r = 0.71, *p* <0.001), and ∆_3-1_ BAI (r = 0.63, *p* <0.001). Similar correlations were observed independently in both sexes.

Changes in whole-body fat mass and fat mass percentage (∆_3-1_ FM kg and ∆_3-1_ FM %) correlated with changes in the same parameters as changes in body weight. Furthermore, correlations between visceral fat level changes (∆_3-1_ VFAT) and the ∆_3-1_ WC, ∆_3-1_ HC, ∆_3-1_ WHR, ∆_3-1_ BAI, ∆_3-1_ WHtR, ∆_3-1_ glucose, ∆_3-1_ insulin, and ∆_3-1_ HOMA-IR were also observed after the weight loss program (Table 6). Similar correlations were observed independently in both sexes. Out of the biochemical parameters, the ∆_3-1_ HOMA-IR was associated with the ∆_3-1_ BW (kg) (r = 0.39, *p* < 0.01, Table 6) and ∆_3-1_ FM kg (r = 0.34, *p* < 0.01 Table 6).

Analyzing the correlation between changes in the HOMA-IR (∆_3-1_) and changes in body weight (∆_3-1_ BW) and fat mass (∆_3-1_ FM) within the male and female groups showed stronger statistical significance correlation in the male (r = 0.58 for ∆_3-1_ BW and r = 0.53 for ∆_3-1_ FM) than female (r = 0.31 for ∆_3-1_ BW and r = 0.32 for ∆_3-1_ FM) participants.

At the end of the WLP (12 weeks), changes in body weight (∆_3-1_ BW) positively correlated with changes in fat mass (∆_3-1_ FATM), fat-free mass (∆_3-1_ FFM), and the visceral fat level (∆_3-1_ VFAT) as well as the ∆_3-1_ HOMA-IR score. When multiple linear regression analysis was used to identify variables that independently predicted the final BW loss (12 weeks), the change in BW after the second visit (∆_2-1_ BW) was identified as a significant predictor (Table 7).

The initial body weight loss (∆_2-1_ BW) appeared to be a better marker of weight loss than the other variable changes at the second visit (∆_2-1_ FATM, ∆_2-1_ FFM, ∆_2-1_ VFAT, and ∆_2-1_ HOMA-IR).

## 4. Discussion

The global epidemic of obesity, defined by the WHO as “globesity”, is continuously growing [46]. Early predictors of effective weight loss are important factors in predicting differences between individuals who start weight loss programs and help identify subjects who will have difficulties achieving expected weight reduction. These subjects would benefit from an individualized therapeutic approach taking into account their specific needs and metabolic rate to help achieve effective weight loss.

In this study, 106 patients that qualified for a 12-week weight loss program (WLP) were characterized in terms of molecular, biochemical, and anthropometric parameters. There were no differences in the measured anthropometric and biochemical parameters at baseline between various genotypes of the *FTO* rs9930506 polymorphism. This result was probably due to the highly homogenized BMI in the study group, which had a narrow range between 30 to 35 kg/m^2^ (class I obesity). However, comparing the prevalence of genotypes and alleles in individuals undergoing the WLP with those in the control group (normal BMI 18.5–24.9 kg/m^2^) showed that the rs9930506 G allele was higher among subjects with obesity (55%) versus those in the control group (44%, OR = 1.49, *p* = 0.014). This corresponds with our previous analyses, which demonstrated that parts of the Polish population carry a genetic variant which, in an obesogenic environment, may significantly enhance the risk of developing obesity [5]. Our study did not demonstrate any significant effects of the unfavorable GG genotype of *FTO* rs9930506 on baseline parameters, inflammation-associated blood mediators (CRP, leptin, and adiponectin), or levels of *IL-6*, *TNF*-*α*, and *NF-κB* mRNA in either WBCs or adipocytes after undergoing the WLP. According to our knowledge, this was the first attempt to find an association of the rs9930506 polymorphism of the *FTO* gene with the level of proinflammatory gene expression before and after body weight change. An analysis of the scientific literature confirms the role of the *FTO* rs9939609 single-nucleotide polymorphism, which comprises the A (the risk allele) and T alleles and has been previously evaluated in relation to proinflammatory marker blood levels [47,48,49]. In 70 Brazilian women with morbid obesity, the effect of genotype on the concentration of IL-6 but not TNF-α in the postprandial period was confirmed [48]. Among 196 overweight Iranian patients of both sexes, the rs9939609 risk allele was associated with higher serum leptin concentrations [49], while among 610 Spanish individuals, no differences were detected among genotype groups in serum adipocytokine (leptin, TNF-α, and IL-6) levels except for adiponectin, the level of which was lower in the TT genotype than the AA genotype group [47].

Significant reductions in risk factors for developing obesity-related diseases are thought to be associated with mild (3–5% of initial weight loss) or moderate (5–10%) weight loss, depending on coexisting complications—greater weight loss leads to greater health benefits [50,51]. In our study, nearly 55% of subjects achieved successful weight loss, i.e., at least 5% (mean 8.9 + 3.1%), as a result of the intervention applied. Among 17.6% of the study individuals a more than 10% weight loss was achieved. Similar to other studies, a 5% weight loss cut-off point was used in further data analysis, as it was felt to be a more realistic therapeutic goal for patients as well as an opportunity for sustainable lifestyle changes, in line with the European Guidelines for Obesity Management in Adults [50]. Our study showed that weight reduction accompanied a statistically significant reduction in body fat after both 4 weeks and 12 weeks of the WLP. In addition, men had higher reductions in initial visceral fat levels and fat-free mass and greater initial weight loss than women.

The relationship between the weight reduction achieved during the 12-week WLP and the selected proinflammatory gene expression levels was assessed in both white blood cells (WBCs) and subcutaneous abdominal adipocytes at several stages in the study. We are reporting for the first time that after 4 weeks of the WLP, the reduction in body weight did not result in a significant decrease in *IL-6*, *TNF-α*, and *NF-κ*B gene expression in WBCs. The analysis also showed no statistically significant changes in expression levels of these genes in WBCs after 12 weeks of the WLP in the whole group nor after subdividing it into two groups based on whether the participants achieved a 5% weight reduction or not. Changes in *NF-κB* gene expression correlated with *IL-6* and *TNF-α* gene expression in subcutaneous adipose tissue (AT) and reflected changes in *NF-κB* and *IL-6* gene expression in the study subjects’ WBCs. However, these results should be confirmed in a larger group of male and female participants to draw any firm conclusions.

Previous studies looking at proinflammatory gene expression in blood and adipocytes among subjects with overweight and obesity versus those with normal weight were assessed in a study by Matulewicz et al. [52]. In contrast to adipose tissue, where the altered expression of the nuclear factor-κB (*NF-κB*) gene was observed in individuals with excessive body weight, no significant differences in mRNA levels of proinflammatory cytokines were found in blood mononuclear cells between the study groups [52]. A further study by the same team of investigators also found no effects of a 12-week dietary intervention (with or without immunomodulatory β-glucan) and an associated mean weight reduction of ~11.3% on the *IL-6* gene expression and other proinflammatory factors in both the subcutaneous abdominal AT and blood mononuclear cells [31]. There was also no effect on proinflammatory genes (i.e., *IL-6* and *TNF-α*) in peripheral monocytes among women with obesity and type 2 diabetes after a short-term (2 weeks) very-low-calorie diet [53]. Our study results were similar to those described above. In contrast, an analysis by de Mello et al. found that the expression of genes (i.e., *IKBKB*, *CCL5*, and *ICAM-1*) involved in *NF-κB* activation was significantly reduced after weight loss (~4.5% BW and 1.5% FM) in patients with metabolic syndrome undergoing dietary therapy [32]. In another report, the same authors showed a relationship between weight loss and a decrease in *TNF-α* mRNA levels (*p* < 0.001) and, interestingly, increased *IL-6* expression (*p* < 0.01) in blood mononuclear cells [33]. The latter result was unexpected and probably related to the pleiotropic effect of interleukin 6, which has pro- and anti-inflammatory effects and is also referred to as the “metabolic hormone” involved in immune responses related to glucose, protein, or lipid metabolism [54]. Our study showed no significant changes in the *IL-6* expression levels in WBCs after 4 weeks and after 12 weeks of the weight loss program. In addition, following 12 weeks of the WLP in subjects with class I obesity, we did not find changes in the expression of the proinflammatory genes (*IL-6*, *TNF-α*, and *NF-κB*) in adipocytes from subcutaneous abdominal tissue occurred earlier than in leukocytes. However, it is known that the adipose tissue in subjects with obesity is dominated by hypertrophic, dysfunctional adipocytes which contribute to the development of local inflammation and take on a systemic function [55]. This complex interplay between adipose tissue fibrosis and hyperplasia and the immune circulating system leads to systemic and metabolic adverse health consequences [56]. In the present study, the group undergoing biopsy (*n* = 20) had a relatively high reduction in body weight (9.0 ± 4.7%) and fat mass (5.5 ± 3.6%) but did not show significant reductions in mRNA levels of *IL-6*, *TNF-α*, and *NF-κB* in adipose tissue after 12 weeks of the WLP. A study by Campbell et al. also reported no association between different weight changes (≤5% vs. 5–10% vs. >10%) and the expression of most inflammatory cytokines, including *TNF-α* and *IL-6*, in subcutaneous adipose tissue [57]. The cited authors indicate that the fat mass reduction effect (~12% FM) in the studied groups was too low to achieve a sufficient effect on the inflammatory process. Similar findings were presented by Strączkowski et al., who postulated that elevated baseline expression levels of genes related to the inflammatory process (i.e., *IL-6*) were not reduced as a result of the 11.3% weight loss achieved by the dietary intervention in individuals with uncomplicated obesity [31]. Changes in the expression of genes involved in inflammation in AT (*IL-6*, *TNF-α*) were not obtained in 35 overweight American subjects of both sexes in the CALERIE study as a result of a 10% reduction achieved in 24 months through a 25% caloric restriction alone or with exercise [55]. In the study by Magkos et al. [27], the clinical effect of progressive weight loss on the reduction in low-grade inflammation—both systemic and localized in subcutaneous adipose tissue—was only observed when body weight was reduced by more than 11% (i.e., ~11.3 kg, obtained after ~27 weeks). Weight loss of 5% over approximately 14 weeks of lifestyle intervention in sedentary lifestyle subjects significantly improved anthropometric parameters and reduced plasma concentrations of some cardiometabolic risk factors (glucose, insulin, and triglycerides) but did not affect changes in subcutaneous adipose tissue gene expression of the proinflammatory cytokines, *IL-6*, and tumor necrosis factor.

Evidence looking at the effects of weight reduction, including the extent of weight reduction, on the downregulation of the gene expression of low-grade inflammatory mediators associated with obesity in adipose tissue, similar to peripheral blood cells, is not consistent. This is certainly related to the different course, nature, duration, and effectiveness of interventions, as well as the differences in the number and type of health problems in the studied patients. Interventions that result in high percentages of weight loss in a relatively short period of time—in the case of interventions involving a very-low-calorie diet (VLCD) [56] or bariatric surgery [58,59,60]—clearly confirm the effect of weight loss on the reduction in the *IL-6* and/or *TNF-α* expression in AT. However, in bariatric patients, this only applies to those with a high initial BMI, most often associated with class III obesity (BMI ≥ 40) [50,51,61,62]. There is speculation that both the degree of weight loss and the effect of weight loss on AT inflammation are largely dependent on the initial degree of obesity, the presence of metabolic disorders [31], or the age of obesity onset [63]. Future research could focus on comparing gene expression results from purified populations of leukocytes or monocytes [64,65], as a study by Whitney et al. [64] found that the proportion of a variety of cell types in peripheral blood is an individual factor and can affect a person’s gene expression profiling results.

The identification of evidence-based predictors for achieving clinically beneficial weight loss is particularly important in the fight against the obesity pandemic due to the numerous comorbidities that accompany obesity. Effective weight reduction is a long-term and multi-stage process. Thus, the predictors of effective body weight loss or body weight regain assessed previously are often not sufficiently substantiated or have a low prediction power [66]. In the present study, changes in BW and fat mass (body fat mass, fat percentage, and visceral fat level) at the end of the WLP were associated with change in the HOMA-IR (homeostatic model assessment for insulin resistance) score, which in the case of BW but not body fat mass was similar to the results in other studies [67]. In addition, we reported that the change in the final HOMA-IR score appears to be a better marker of metabolic improvement than other variables studied, such as triglycerides, total cholesterol, HDL cholesterol, LDL cholesterol, CRP, adiponectin, and leptin. Similar to our results, Bozkuş et al. found that a change in the HOMA-IR correlated with change in body weight and BMI at the end of a short-term intervention (12 weeks) [67], while the reduction in LDL cholesterol and the elevation in HDL cholesterol levels were not statistically significant. As shown elsewhere, the HOMA-IR score changes after short-term interventions were predictive of weight changes 6 months after the end of the intensive weight loss phase [10]. The HOMA-IR is considered primarily as a parameter which helps the assessment of insulin resistance (IR). Its strong correlation with results from the gold standard, a euglycemic clamp, are believed to be representative and helpful in determining peripheral IR [12,68]. In the present study, correlations with changes in most of the measured variables (BW, BMI, FM, VFAT, WC, WHtR, and BAI) and changes in the HOMA-IR score were found. The changes in glucose and insulin concentration were also positively correlated with changes in BW and FM. A predictive relationship between weight loss and changes in plasma glucose levels in patients with obesity and type II diabetes has been previously indicated [69].

In a linear regression model, we identified the initial change in BW (after 4 weeks) as the strongest predictor of success of final weight loss (after 12 weeks of the WLP). This finding is consistent with earlier reports in which greater initial weight loss was one of the strongest short-term, mid-term, and long-term predictors of successful weight loss outcomes [66,70,71,72,73,74]. In the NUGENOB project, early (1 week) and half-way (5 week) weight loss was found to be significantly correlated with final weight loss among study participants from seven European countries undergoing a 10-week intervention based on a hypo-energetic diet (a 4 kg weight reduction at week 5 was the strongest predictor of a 10% weight loss at week 10) [71]. In another European study, the DIOGENES study, early weight loss (at both week 1 and week 3 of a low-calorie diet) showed a strong predictive power for final weight loss (at week 8) [72]. Similarly, in Swedish patients with obesity, early weight loss played a strong predictive role for a subsequent weight loss effect [70]. Our observations in the context of similar studies discussed above indicate that detecting early changes in weight loss can have a significant effect on helping patients manage weight loss more effectively, including a better final response to weight loss therapy among individuals with obesity.

There were several weaknesses in the present study. First, it included a limited number of patients with class I obesity in the analyses. Secondly, there was an imbalance between the number of male and female participants. Furthermore, we were unable to analyze the effects of a dietary intervention on proinflammatory gene expression in white blood cells (WBCs) and subcutaneous adipose tissue (AT) according to sex, due to low participant numbers. In our study, insulin resistance was measured using the HOMA algorithm, not a euglycemic clamp. However, this method is widely known and is readily available in everyday practice as a predictive factor in assessing the effectiveness of weight reduction. We believe that an interesting direction for further analysis which can enhance the baseline characteristics of patients with obesity would include collecting psychosocial or environment data. Tools used in health promotion and psychology, such as The Generalized Self-Efficacy Scale (GSES) and The Multidimensional Health Locus of Control Scale (MHLC), may be helpful and valuable in gaining additional patient characteristics in future research. These additional results could be analyzed with respect to the *FTO* gene genotype.

In conclusion, initial weight loss associated with a significant increase in insulin sensitivity appears to be a valuable indicator of weight loss success and an important aspect of individualized therapeutic approaches in clinical practice. The *FTO* rs9930506 genotype polymorphism was not found to determine weight loss success or the reduction in levels of inflammation markers. After 4 and 12 weeks of the WLP, the reduction in body weight did not result in a significant decrease in *IL-6*, *TNF-α*, and *NF-κB* gene expression in WBCs or subcutaneous AT.

## Figures and Tables

**Table 1:** Assay information.

Gene Name	Gene Symbol	Assay
Interleukin 6	*IL-6*	Hs00985639_m1
Tumor necrosis factor α	*TNF-α*	Hs01113624_g1
Nuclear factor kappa B subunit 1	*NF-κB*	Hs00765730_m1
Eukaryotic 18S rRNA	*18S*	Hs99999901_s1

**Table 2:** The baseline characteristics of patients with BMI ≥ 30 kg/m^2^ according to the genotype of *FTO* rs9930506.

		*FTO* rs9930506 Polymorphism			
	Total (*n* = 106)	GG (*n* = 32)	AG (*n* = 52)	AA (*n* = 22)	ANOVA/Kruskal–Wallis Tests *p*
Variables	Mean ± SD	Mean ± SD	Mean ± SD	Mean ± SD	
Age (years)	38.7 ± 5.5	38.5 ± 5.2	39.0 ± 5.8	38.4 ± 5.5	0.860
Calorie intake (kcal)	2125.6 ± 771.0	2284.2 ± 832.7	2036 ± 648.9	2079.1 ± 924.4	0.399
Weight (kg)	94.13 ± 13.5	93.6 ± 12.3	94.6 ± 14.1	93.8 ± 14.3	0.932
BMI	32.6 ± 2.3	32.0 ± 2.4	32.3 ± 2.4	32.5 ± 2.1	0.553
WC (cm)	104.3 ± 9.3	103.7 ± 8.1	103.5 ± 10.1	107.4 ± 8.1	0.248
HC (cm)	114.0 ± 6.7	112.4 ± 6.7	114.6 ± 6.4	114.9 ± 7.0	0.252
Fat (%)	34.9 ± 4.9	34.1 ± 5.3	35.0 ± 4.8	35.7 ± 4.6	0.496
VFAT (scores)	9.5 ± 3.1	9.3 ± 3.2	9.7 ± 3.3	9.2 ± 2.7	0.770
BAI	33.8 ± 4.7	33.0 ± 5.6	34.2 ± 4.4	34.0 ± 4.2	0.519
WHtR	0.61 ± 0.04	0.61 ± 0.04	0.61 ± 0.04	0.63 ± 0.04	0.103
Glucose (mg/dL)	88.4 ± 7.3	87.8 ± 6.9	88.6 ± 8.1	88.8 ± 6.2	0.867
Insulin (µIU/mL)	12.5 ± 6.3	11.0 ± 5.2	13.1 ± 7.5	13.3 ± 4.2	0.270
HOMA-IR	2.8 ± 1.6	2.4 ± 1.1	2.9 ± 2.0	3.0 ± 1.1	0.238
Total cholesterol (mg/dL)	195.2 ± 36.6	191.0 ± 38.0	198.8 ± 33.3	193.0 ± 42.6	0.610
Triglycerides (mg/dL)	135.2 ± 65.6	126.1 ± 60.3	138.7 ± 73.5	140.2 ± 53.9	0.646
hs-CRP (g/L)	2.8 ± 3.1	2.2 ± 1.8	2.6 ± 2.8	4.0 ± 4.8	0.211
Leptin	20.7 ± 6.9	23.2 ± 9.7	19.6 ± 5.2	19.8 ± 5.2	0.130
Adiponectin	6078.0 ± 2166.1	6412.2 ± 2553.5	5883.1 ± 2082.3	6011.7 ± 1901.3	0.756

The values are given as means ± standard deviations (SD). Abbreviations: BMI, body mass index; WC, waist circumference; HC, hip circumference; BAI, body adiposity index; WHtR, waist to height ratio; VFAT, visceral fat level in 1–18 scores estimated by BIA (bioelectrical impedance analysis); HOMA-IR, homeostatic model assessment for insulin resistance; CRP, C-reactive protein; *p* values were considered significant when *p* < 0.05.

**Table 3:** Genotype distribution and allele frequency of the *FTO* rs9930506 polymorphism in the studied group and controls with BMI 18.5–24.9 kg/m^2^.

*FTO* rs9930506 (A/G)				
Groups	Genotypes *n* (%)			
	AA	AG		GG
Studied groups	22 (21%)	52 (49%)		32 (30%)
Controls	74 (28%)	148 (55%)		46 (17%)
Co-dominant model OR (95% CI); *p*	1.54 (1.10–2.16); 0.011			
Groups	Genotypes *n* (%)			
	GG		AA + AG	
Studied groups	32 (30%)		74 (70%)	
Controls	46 (17%)		222 (83%)	
Recessive model OR (CI); *p*	2.09 (1.24–3.52); 0.006			
Groups	Genotypes *n* (%)			
	GG + AG		AA	
Studied groups	84 (79%)		22 (21%)	
Controls	194 (72%)		74 (28%)	
Dominant model OR (CI); *p*	1.46 (0.85–2.50); 0.165			
Groups	Alleles *n* (frequency)			
	A		G	
Studied groups	96 (0.45)		116 (0.55)	
Controls	296 (0.55)		240 (0.45)	
Additive model OR (CI); *p*	1.49 (1.08–1.42); 0.014			

A = wild; G = polymorphic; OR: odds ratio; CI: 95% confidence interval for the OR.

**Table 4:** Differences in changes in selected parameters between individuals with higher (≥5%) and lower (<5%) weight loss during 12 weeks of weight loss program (WLP).

Variables	Group A (*n* = 40) Weight Loss ≥ 5%	Group B (*n* = 37) Weight Loss < 5%	*p* Value by Effect *	*p* Value by Sex **	*p* Value by Effect and Sex ***
BW (kg)					
∆_2-1_ (4-week)	−4.8 ± 2.3 **	−2.1 ± 1.2	<0.001	0.139	0.014
∆_3-1_ (12-week)	−8.3 ± 3.0	−2.5 ± 1.9	<0.001	0.031	0.188
BMI (kg/m^2^)					
∆_2-1_ (4-week)	−1.7 ± 0.8	−0.7 ± 0.4	<0.0001	0.571	0.142
∆_3-1_ (12-week)	−2.9 ± 1.0	−0.9 ± 0.7	<0.0001	0.251	0.951
WC (cm)					
∆_2-1_ (4-week)	−4.0 ± 2.3	−2.4 ± 2.3	0.014	0.296	0.244
∆_3-1_ (12-week)	−7.9 ± 3.1	−3.0 ± 3.6	<0.0001	0.251	0.248
BAI					
∆_2-1_ (4-week)	−1.3 ± 0.8	−0.9 ± 0.8	0.103	0.098	0.585
∆_3-1_ (12-week)	−2.5 ± 1.2	−1.4 ± 1.4	<0.001	0.549	0.142
WHtR					
∆_2-1_ (4-week)	−0.02 ± 0.01	−0.01 ± 0.01	0.016	0.521	0.371
∆_3-1_ (12-week)	−0.05 ± 0.02	−0.02 ± 0.02	<0.001	0.280	0.928
FM (kg)					
∆_2-1_ (4-week)	−2.9 ± 1.6	−1.5 ± 1.4	<0.001	0.635	0.241
∆_3-1_ (12-week)	−5.4 ± 2.7	−1.6 ± 1.8	<0.0001	0.092	0.578
VFAT					
∆_2-1_ (4-week)	−0.9 ± 0.7	−0.3 ± 0.6	<0.001	<0.001	0.001
∆_3-1_ (12-week)	−1.6 ± 1.0	−0.5 ± 0.7	<0.001	<0.001	0.004
FFM (kg)					
∆_2-1_ (4-week)	−2.0 ± 1.7	−0.5 ± 1.5	<0.001	0.118	0.040
∆_3-1_ (12-week)	−2.8 ± 1.7	−0.7 ± 1.5	<0.0001	0.097	0.149
∆ HOMA-IR					
∆_2-1_ (4-week)	−0.9 ± 1.5	−0.3 ± 1.1	0.136	0.234	0.330
∆_3-1_ (12-week)	−0.9 ± 1.6	0.2 ± 2.1	0.026	0.244	0.555

Abbreviations: ∆, change (final–baseline); BW, body weight; BMI, body mass index; WC, waist circumference; BAI, body adiposity index; WHtR, waist to height ratio; FM, fat mass; VFAT, visceral fat level in 1–18 scores estimated by BIA (bioelectrical impedance analysis); FFM, fat-free mass. Data described as mean ± SD; *p* value < 0.05 was considered statistically significant. * Difference between group A and B; ** difference between women (*n* = 59) and men (*n* = 18) in entire group (*n* = 76); *** difference in parameter changes between women (*n* = 28) and men (*n* = 12) in group A.

**Table 5:** Correlations for changes in the *IL-6*, *TNF-α*, and *NF-κB* mRNA levels in patients’ white blood cells (WBCs) and subcutaneous adipose tissue (AT) in response to the weight loss program.

∆_3-1_	∆_3-1_ AT *IL-6*	∆_3-1_ AT *TNF-α*	∆_3-1_ AT *NF-κB*
WBCs *IL-6*	0.16	0.15	−0.42
WBCs *TNF-α*	0.69	0.65	0.51
WBCs *NF-κB*	0.89 ***	0.35	0.75 *
AT *IL-6*	-	0.65 *	0.84 ***
AT *TNF-α*	0.65 *	-	0.85 ***
AT *NF-κB*	0.84 ***	0.85 ***	-

Abbreviations: ∆, change (3rd visit–1st visit). Data expressed as correlation coefficient; *p* value (*** *p* < 0.001, * *p* < 0.05).

**Table 6:** The relationship between changes in anthropometric and biochemical factors after the 12-week weight loss program.

∆_3-1_	∆_3-1_ BW (kg)	∆_3-1_BMI (kg/m^2^)	∆_3-1_ FM (%)	∆_3-1_ FM (kg)	∆_3-1_ VFAT (Scores)
WC (cm)	0.72 ***	0.72 ***	0.62 ***	0.72 ***	0.67 ***
HC (cm)	0.54 ***	0.58 ***	0.51 ***	0.57 ***	0.47 ***
WHR	0.44 ***	0.42 ***	0.35 **	0.42 ***	0.41 ***
WHtR	0.71 ***	0.72 ***	0.62 ***	0.71 ***	0.65 ***
BAI	0.63 ***	0.67 ***	0.53 ***	0.60 ***	0.46 ***
Triglycerides (mmol/L)	0.13	0.09	0.11	0.08	0.17
Total cholesterol (mg/dL)	0.07	0.07	0.12	0.10	0.07
HDL cholesterol (mg/dL)	0.18	0.20	0.14	0.18	0.04
LDL cholesterol (mg/dL)	0.02	0.03	0.07	0.05	0.01
Glucose (mg/dL)	0.26 *	0.26 *	0.27 *	0.28 *	0.26 *
Insulin (mU/L)	0.39 **	0.35 **	0.24 *	0.32 **	0.31 **
HOMA-IR	0.39 **	0.36 **	0.24 *	0.34 **	0.33 **
CRP (mg/L)	−0.02	−0.03	−0.20	−0.12	0.07
Adiponectin (ng/mL)	−0.25	−0.20	−0.17	−0.19	−0.26
Leptin (ng/mL)	−0.16	−0.18	−0.07	−0.08	−0.13

Abbreviations: ∆_3-1_, change (3rd visit–1st visit); BW, body weight; BMI, body mass index; FM, fat mass; VFAT, visceral fat level in 1–18 scores estimated by BIA (bioelectrical impedance analysis); WC, waist circumference; HC, hip circumference; WHR, waist–hip ratio; BAI, body adiposity index; WHtR, waist-to-height ratio; HDL, high-density lipoprotein; LDL, low-density lipoprotein; HOMA-IR, homeostatic model assessment for insulin resistance; CRP, C-reactive protein. Data expressed as correlation coefficient; *p* value (*** *p* < 0.001, ** *p* < 0.01, * *p* < 0.05).

**Table 7:** Regression analyses showing associations of initial body weight change (∆_2-1_ BW) and change in body weight (∆_3-1_ BW) after 12 weeks of weight loss program.

	∆_3-1_ BW			∆_3-1_ BW			∆_3-1_ BW		
Variables	Model 1			Model 2			Model 3		
	β	95% CI	*P*	β	95% CI	*P*	β	95% CI	*P*
∆_2-1_ BW	0.81	0.65–0.96	0.000	0.80	0.38–1.21	0.000	0.70	0.22–1.18	0.000

Model 1: adjusted for age, sex, and baseline physical activity. Model 2: adjusted for ∆_2-1_ FATM, ∆_2-1_ FFM, and ∆_2-1_ VFAT. Model 3: adjusted for Model 2 and ∆_2-1_ HOMA-IR. Abbreviations: BW, body weight; CI, confidence interval; β, coefficient for changes in body weight ∆_3-1_ BW at 3rd visit (after 12 weeks of the weight loss program).

## Data Availability

The data that support the findings of this study are available from the corresponding author upon reasonable request.
